# Motor Skills and Executive Functions in Pediatric Patients with Down Syndrome: A Challenge for Tailoring Physical Activity Interventions

**DOI:** 10.3390/pediatric15040062

**Published:** 2023-11-10

**Authors:** Matteo Vandoni, Matteo Giuriato, Agnese Pirazzi, Sara Zanelli, Francesca Gaboardi, Vittoria Carnevale Pellino, Alessandra Anna Gazzarri, Paola Baldassarre, Gianvincenzo Zuccotti, Valeria Calcaterra

**Affiliations:** 1 Laboratory of Adapted Motor Activity (LAMA), Department of Public Health, Experimental Medicine and Forensic Science, University of Pavia, 27100 Pavia, Italy; matteo.vandoni@unipv.it (M.V.); matteo.giuriato@unipv.it (M.G.); agnese.pirazzi01@universitadipavia.it (A.P.); vittoria.carnevalepellino@unipv.it (V.C.P.); 2 Pediatric Department, “V. Buzzi” Children’s Hospital, 20154 Milan, Italy; sara.zanelli01@universitadipavia.it (S.Z.); francesca.gaboardi@unimi.it (F.G.); gianvincenzo.zuccotti@unimi.it (G.Z.); 3 Department of Industrial Engineering, University of Rome Tor Vergata, 00133 Rome, Italy; 4 Associazione Vivi Down Onlus, 20158 Milan, Italypaola.baldassarre@unimi.it (P.B.); 5 Department of Biomedical and Clinical Science “L. Sacco”, University of Milan, 20157 Milan, Italy; 6 Pediatric and Adolescent Unit, Department of Internal Medicine, University of Pavia, 27100 Pavia, Italy

**Keywords:** Down syndrome, motor skills, executive functions, children, adolescents, physical activity

## Abstract

Down syndrome (DS) is one of the most common chromosomal disorders. In addition to this variety of dysmorphic features. DS is also associated with a wide range of diseases and related comorbidities affecting different organs and systems. These comorbidities, together with societal and environmental influences, have a negative impact on physical activity in people with DS. Low levels of physical activity and energy expenditure have been identified as crucial players in worsening the acquisition of motor skills and executive functions. Executive functions are critical for the many skills (creativity, flexibility, self-control, and discipline) impacting our quality of life and make it possible to control impulses, mentally play with ideas, and stay focused. We proposed a broad overview of the available literature regarding motor skills and executive functions in pediatric patients with DS to understand the specific challenges for tailoring physical activity interventions. Motor skill interventions are effective in improving motor competence and performance on cognitive, emotional, and physical aspects in children with DS. Interventions based on executive functions in DS subjects are effective to contrast the cognitive decline and improve the everyday use of executive functions in youth and adults. Targeted interventions are mandatory for maximizing the benefits of physical activity, minimizing potential risks, and ultimately improving the overall health outcomes and quality of life for individuals with DS.

## 1. Introduction

Down syndrome (DS) is one of the most common chromosomal disorders. The incidence of DS is between 1/700 and 1/800 live births worldwide [1,2], and the average life expectancy of affected patients has increased substantially, from 25 years in 1983 to 60 years in 2020 [3].

DS is caused by trisomy of chromosome 21. In 95% of cases [4], there is a complete trisomy 21, resulting mainly from an error in maternal meiosis. Advanced maternal age at conception (>35 years) is the major risk factor which can determine non-disjunction of homologous chromosomes or chromatids during the meiotic divisions [1,2]. A partial trisomy 21 is possible but not frequent; in this case, symptoms vary according to the length of the triplicated portion [5]. About 5% of cases are determined by a translocation, mainly t(14;21) or t(21;21). Mosaicism occurs only in 2% of the children affected. The gene content of chromosome 21 is currently estimated to comprise 329 genes, and some of them seem to participate in mitochondrial energy generation and reactive oxygen species metabolism [6].

DS is the most frequent cause of genetic intellectual disability. Individuals with DS show typical phenotypic features, such as hypotonia, brachycephaly, flat nasal bridge, epicanthal folds, brachydactyly, fifth finger clinodactyly, lax ligaments, and sandal gap [2,5,7]. In addition to this variety of dysmorphic features, DS is also associated with a wide range of diseases and related comorbidities affecting different organs and systems [7].

**Figure 1:**
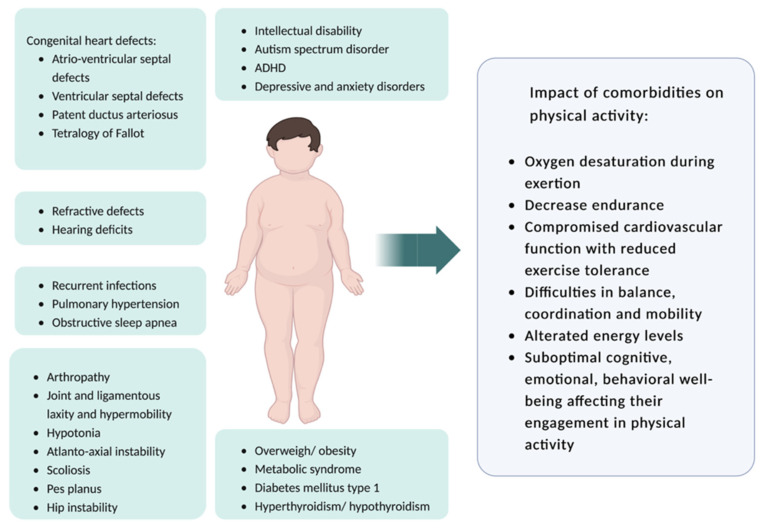
Comorbidities with a negative impact on physical activity in patients with Down syndrome (created with Biorender https://www.biorender.com, accessed on 1 August 2023).

These comorbidities, together with societal and environmental influences, such as competing family responsibilities and a lack of accessible programs [8], have a negative impact on physical activity (PA), contributing to a sedentary lifestyle in people with DS. In particular, (1) cardiac abnormalities (such as atrioventricular septal defects and ventricular septal defects) can lead to compromised cardiovascular function and reduced exercise tolerance [2]; (2) respiratory problems (such as recurrent respiratory tract infections, obstructive sleep apnea, and pulmonary hypertension [4,5]) can hinder physical activity participation and contribute to exercise-related difficulties (such as decreased endurance and oxygen desaturation during exertion); (3) musculoskeletal abnormalities (such as joint laxity, decreased muscle tone (hypotonia), and ligamentous laxity) may impede mobility, balance, and coordination, making PA challenging, requiring strict limitations on certain physical activities, and could prove other findings (such as atlantoaxial instability) to be life threatening [9]; (4) obesity [9,10] can impact overall physical performance; (5) thyroid disorders that can impact energy levels and metabolism [11,12]; (6) altered hormonal adaptations during exercise (such as increased resting leptin and insulin, decreased insulin sensitivity, altered catecholamines responses, decrease of cortisol and testosterone at rest and in response to exercise, and decreased fat oxidation ability) can cause fat mass development and reduced endurance capacity, leading to exercise limitations [13]; and (7) neuropsychiatric disorders (such as autism spectrum disorder, attention deficit hyperactivity disorder, and depressive/anxiety disorders) can impact their cognitive, emotional, and behavioral wellbeing, affecting their engagement in physical activity [12,14]. The comorbidities associated with a negative impact on PA in patients with DS are resumed in Figure 1.

Low levels of PA and energy expenditure have been identified as a growing concern within the DS population and represent a crucial factor that can worsen the acquisition of motor skills and executive functions (EFs). Understanding the motor consequences of sedentary behavior in individuals with DS is essential for developing effective interventions and promoting overall health and wellbeing.

We proposed a broad overview of the available literature regarding motor skills and executive functions in pediatric patients with DS to understand the specific challenges for tailoring physical activity interventions. Engaging in regular exercise can improve cardiovascular fitness, muscle strength, motor skills, cognitive function, and psychological wellbeing. However, targeted interventions are mandatory to maximize the benefits of physical activity and minimize potential risks, ultimately improving the overall health outcomes and quality of life for individuals with DS.

## 2. Methods

We performed a narrative review [15], presenting a non-systematic summation and analysis of the available literature on the topic of motor skills and executive functions in pediatric patients with DS, with a focus on the pediatric age. We considered the most relevant original scientific papers, clinical trials, meta-analyses, and reviews published until 15 July 2023, in the English language. Case reports and case series were excluded. For this purpose, we carried out a search for relevant papers using three databases (MEDLINE/PubMed, Scopus, and Web of Science) using specific keywords (alone or combined), such as motor skills, executive functions, Down syndrome, children and adolescents, and physical activity.

**Figure 2:**
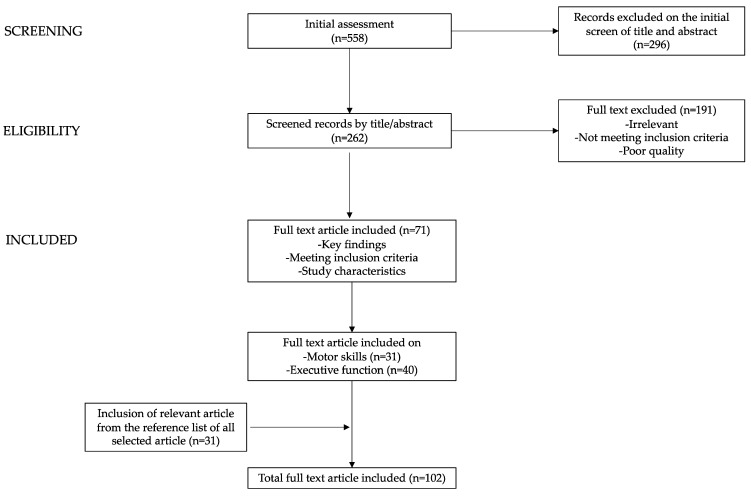
Graphical diagram showing the process of the selection and exclusion of the manuscripts used in writing this narrative review.

Starting from a total of 558 papers, the authors assessed the titles and abstracts (n = 262) and consequently reviewed the full texts of relevant studies (n = 71; motor skills n = 31; executive functions n = 40). The reference list of all manuscripts was also considered to identify relevant manuscripts (n = 31). In Figure 2, the process of the selection and exclusion of papers is shown.

## 3. Motor Skills in Pediatric Patients with Down Syndrome

Motor skills are crucial for our physical activity, survival, and development. In fact, effective motor skills are important for children’s physical, social, and psychological development [16]. Preschool age is decisive for the development of motor skills, and it is considered the most promising time window in relation to preventive strategies based on improved motor skills.

Consequently, there is evidence of many health benefits that can be gained from an improvement in motor skills. For instance, it has been demonstrated that good motor skills positively influence cardiorespiratory fitness and body weight as well as sports participation [16], with all studies suggesting that early competency in motor skills may have important health implications. Furthermore, there are indications of relationships with language development [14,17], executive function [18], and general wellbeing. Since there is a strong interaction between motor skills and cognitive benefits, motor skills training promotes cognitive efficiency in children and adolescents [19]. In addition, cognitive brain function develops to regulate the learning and control of motor skills [20].

Children with DS tend to have lower levels of PA practice compared to their healthy peers and, consequently, are at a higher risk to become overweight obese and suffer from detrimental effects on health-related outcomes. Sedentary behavior and excessive fat mass exacerbated the risk of developing co-morbidities and mortality. In children with DS, lower PA levels and overweight/obesity conditions contribute to a decrease in the acquisition of motor skills [15].

Motor skills refer to the ability to control and coordinate movements of the central nervous system and the muscles of the body, and are essential for performing various PAs, from basic actions (such as walking and grasping objects) to more complex tasks (such as participating in sports). For these reasons, motor skills are typically categorized into two main types: gross motor skills (GMS), which involve the use of larger muscle groups and are related to activities that require strength, balance, and coordination of the whole body; and fine motor skills (FMS), which involve the use of smaller muscle groups, particularly those in the hands and fingers, and are related to activities that require precise control and dexterity. Therefore, motor skills improve over time through practice and experience and are crucial for everyday tasks.

In children, the development of motor skills is a crucial aspect of their overall growth and cognitive development because they often experience delays in the development of their motor skills (such as sitting, crawling, standing, and walking), which are caused by a lower muscle tone (hypotonia) and weaker core strength, and can affect their ability to control their bodies and movements. Difficulties with FMS can impact their ability to perform self-care tasks, such as dressing and feeding themselves. Adapted PA can help children with DS to improve both GMS and FMS through specific activities that promote physical capacities, hand–eye coordination, finger dexterity, and hand strength, which are all movements that mechanically activate both hemispheres of the brain through the motor and sensory cortexes [21]. Exercise can stimulate the brain in such a way that neurons are often in a condition to handle different data from the outside environment, transforming them into the required task [22]. In recent decades, research has shown that the proficiency in fundamental movement skills is important for promoting the physical (i.e., cardiorespiratory fitness, healthy weight) and psychosocial (i.e., physical self-concept) health of children [21].

The GMS of children with DS are consistently low compared to those of normal children, and the largest difference is seen in relation to balance [23]. Balance is one of the major factors that affect the safety and independence skills of children with DS, and is the most difficult function to acquire. Dysfunction in balance may lead to difficulties in psychomotor abilities [24], especially those that are more advanced in childhood motor development [24]. Balance and motor functions are correlated; thus, both aspects of development should be considered together when designing a PA program for children with DS [25].

Furthermore, children with DS exhibit low muscle tone and have less muscular tension. This condition forces one to exert more effort during activities that are needed to activate muscle mass. The consequence, due to the extra effort required, is difficulty in maintaining postural stability and fatigue setting in almost immediately [26]. Therefore, children with DS often display decreased muscle strength, decreased activity tolerance and endurance, and a rounded shoulder posture [26]. These physical factors contribute to a reduced ability to sustain a proper posture necessary to the demands of activities. In addition, hypermobility is another component of low muscle tone; ligaments hold the joints together and they are characterized by slackness and are easily stretched [27]. These lead to excessive flexibility and range of movement, with a limitation in children’s motor control over their movements. Since retarded motor functions may cause delays in acquiring abilities in areas of development (such as mental, emotional, and social), gross and fine motor skills training in children with DS have to be applied appropriately with proper/right stimuli [24]. Thus, many people with DS often choose sedentary ways of life or activities, which attributes them to being sedentary, not having enough energy, or having too much skill [28].

In addition, Chen et al. have examined the balance of people with DS by analyzing their center of pressure (COP); in their analysis, children with DS performed the standing reach test with an increased reaction and execution time and decreased amplitudes of the COP displacements [29]. For this reason, individuals with DS use inefficient compensatory strategies (such as increasing their step width, increasing the frequency of mediolateral COP displacement, decreasing anteroposterior displacement, and increasing trunk stiffness and posterior trunk displacement to maintain equilibrium) [30].

There is also a relationship between balance and GMS and FMS in children with DS, as demonstrated in the study by Wang et al., which found that better GMS in the standing (walking) position and greater muscular strength were associated with smaller COP displacements in both mediolateral and anteroposterior directions during static standing [31]. Three areas of motor ability, including standing motor skills, walk/run/jump motor skills, and muscle strength, were found to significantly contribute to the displacement and velocity of postural sway during voluntary movements [32]. Furthermore, GMS are the foundation for daily routine movements, physical activities, and sports. From a health perspective, higher levels of GMS are associated with a lower body mass index, better cardiorespiratory fitness and PA, as well as enhanced cognitive development, social development, and language skills [33].

Adapted PA programs and activities are used to improve lower limb muscle strength and postural control in children with DS, including progressive resistance training, hippotherapy, aquatic therapy, and isokinetic strength training [34]. Research evidence supports hippotherapy as an effective recognized intervention for GMS training. Furthermore, Azab et al. demonstrated that twelve weeks of trampoline-based stretch–shortening cycle exercises are likely effective for enhancing muscle strength and postural control in children with DS and should consequently be included in the rehabilitation programs for these children. They should be included on the basis that the stretch–shortening cycle exercises are a traditional form of resistance training where the muscles moves rapidly through the eccentric, isometric, and concentric phases [35]. Different models of training were tested, and the water-based PA method by Naczk et al. demonstrated that a 33-week swimming program significantly improved the health status and swimming skills in adolescents with DS [36]. In fact, specific exercise training programs improve the strength and balance in children with DS. Gupta et al. showed the positive adaptation of progressive resistance exercises for the lower limbs and exercises for balance training over a period of six weeks, three times a week [37]. Also, A Eid et al. corroborate the beneficial role of physical exercise in children with DS, analyzing that isokinetic training (3 days a week for 12 weeks) improves postural balance control [26]. Positive results were found in a study by Saeed Alsakhawi, which demonstrated that treadmill training in children with DS is a scheduled and typically prescribed physical therapy intervention that aims to reduce the delay in the onset of walking [38]. In fact, treadmill exercises stimulate the kinetic, kinematic, and temporal features of walking [39]. These exercises improve the strength of the muscles of the lower extremities, enhance motor learning, improve functional abilities, and activate the locomotor control system.

For these reasons, GMS are fundamental to perform functional activities and to participate in fine motor tasks [40]. In fact, children with DS have difficulties with FMS due to their low muscle tone and/or hypermobility in their hands, wrist, or elbows [40]. Instability in the hands makes it much harder for children to tackle higher-level fine motor activities, such as using zips and buttons or cutting. Tactile perception, postural control, bilateral coordination, and dexterity are the building blocks of fine motor skills [41]. Therefore, small movement of the hands, wrists, and fingers have to be trained to develop good FMS; also, good core stability and strength in shoulders and arms is necessary [42]. Upper limb strength provides a stable base for the hands; however, a lack of strength and stability causes difficulties in children to develop their FMS, which are more impaired than GMS. Consequently, they are characterized by lower developmental increases because they refer to the precision, dexterity, and coordination of the hands [43]. Hence, bilateral coordination, pinch and grip strength, separation of the sides of the hands, arch development, finger isolation, thumb web space, and others, are the developmental areas that need to be stimulated to train the use of the fingers and hands [43].

To sum up, low muscle tone, decreased strength, overly flexible joints, and difficulty with balance contribute to reduced postural control. For children with DS, these physical factors cause difficulties in stabilizing and reaching the demands of certain activities [44]. Protocols for strength and balance training should include horizontal jumps, vertical jumps, one-leg stances with eyes open, tandem stances, walking on lines, walking on balance beams, and jumping on a trampolines [25]. Instead, results of strength training have large to moderate effects on general strength, moderate to small effects on maximal strength, and small effects on functional mobility tasks when neuromuscular training is used [26].

## 4. Executive Functions in Children and Adolescents with Down Syndrome

EFs have been defined as a family of top-down mental processes which are necessary for focusing and paying attention to certain stimuli when our instincts or intuitions are inappropriate or inadequate in response to a peculiar situation [45]. EFs have been shown to be crucial for physical and mental health, for appropriate outcomes in life and school, and for social, cognitive, and psychological growth [46]. In general, EFs have been divided into three basic groups [47]: inhibition, working memory, and cognitive flexibility. In particular (when necessary), inhibitory control allows people to consciously redirect attention, actions, thoughts, and emotions. On the contrary, lack of inhibitory control results in a susceptibility to impulses, ingrained habits, and external temptations. It allows to make deliberate choices and avoid thoughtless reactions [46]. Early life inhibitory control strongly predicts lifelong outcomes. Moffitt et al.’s results showed that better inhibitory control in children from ages 3 to 11 (showing patience, focus, persistence, and reduced impulsivity) had more positive outcomes in comparison to teenagers and adults. People with greater inhibitory control were more likely to stay in school, make responsible choices, and refrain from risky behaviors, such as smoking or drug use [48]. Inhibitory control usually tends to decline significantly with normal aging [46].

Working memory is another key component of EFs and involves the capacity to hold information in mind and manipulate it mentally. It can be divided in two main models based on the content: verbal and nonverbal (visual-spatial). Working memory plays a crucial role in comprehending anything that unfolds sequentially, as it requires retaining what happened earlier and connecting it to subsequent events. This ability is fundamental for understanding written or spoken language, from sentences to longer passages, performing mental calculations, reordering items in your mind (such as organizing a to-do list), translating instructions into action plans, updating your thinking with new information, considering alternatives, and drawing general principles or connections between ideas [46]. The ability to retain information in working memory starts developing at a very young age, with infants and young children demonstrating the capacity to hold one or two items in memory for extended periods [49]. As individuals age, working memory tends to decline; this decline is attributed to reduced inhibitory control in older adults, making them more susceptible to proactive and retroactive interference [50] as well as distractions [51]. Finally, cognitive flexibility is a core component of EFs that involves the ability to adapt thought, strategies, or mental processes in response to changing circumstances, new information, or different tasks. It enables individuals to shift their cognitive focus, switch between tasks, generate alternative solutions to problems, and adjust their behavior or plans as needed [47].

EFs are also classified as “hot” or “cool” in relation to the complexity degree or the emotional and affective engagement of the task performed [52]:

Hot EFs, such as emotional control and inhibition, are associated with the ventromedial prefrontal cortex, and consequently with affection and motivation. The ventromedial prefrontal cortex is connected to the hypothalamus, thalamus, and limbic structures that drive the effective evaluation of behaviors.

Cool EFs, such as working memory, planning and organizing, and cognitive flexibility, are related to the dorsolateral prefrontal cortex. This is stimulated by cognitive demands and selective information related to memory tasks, and retains operational memory to plan appropriate actions over specific objectives. Therefore, since it is involved in both hot and cool EFs, the prefrontal cortex is the main region related to EFs and leads multiple processes, including cognition, behavior, language, and reasoning [53].

A reduction in the volume of the prefrontal cortex, limbic system, and brain stem has been related to subjects with DS [54]. Indeed, a deficit in EF tasks has been described in several developmental disorders, such as DS [55]. In the light of this, children with DS tend to demonstrate more pronounced difficulties in EFs, particularly in working memory tasks that demand a significant level of cognitive control when compared to typically developing children with a similar mental age [55]. Additionally, children with DS often display weaker response inhibition skills and struggle with concurrent cognitive tasks [56].

In particular, in subjects with DS, there do not appear to be significant differences compared to typically developing controls in inhibition and fluency, indicating that these skills develop in alignment with overall cognitive development [57]. However, the presence of difficulties in verbal and visuospatial executive working memory suggests a potential delay or abnormal development in this specific domain, which can impact the everyday tasks that require concurrent storage and processing [57]. It is important to note that the educational implications of working memory difficulties may be significant, as these skills are known to be correlated with progress in reading, spelling, and mathematics in typically developing children. Furthermore, the presence of difficulties in verbal set shifting in DS may be influenced by the complexity of tasks, suggesting that specific verbal executive deficits may become more apparent when tasks become more demanding [57].

Moreover, accelerated volume loss in subjects with DS is observed in the frontal, temporal, and parietal lobes, and this leads to cognitive declines in middle to late adulthood, which are frequently related to dementia, while neurodegenerative changes can take place even in the absence of clinical signs of dementia [58]. There is also evidence of reduced connectivity, reflecting a decreased capacity to integrate information from remote areas of the brain into consistently distributed networks [59]. As a result, children with DS show impairments in many traits of attention (auditory sustained attention, visual selective attention) that extend beyond those expected for their mental age [36]. They also show difficulty executing a strategy to problem solve and execute actions in planning, even though shifting mental sets is particularly challenging for children and adults with DS [55].

In addition, over the years, adults with DS have been affected by cognitive deficits, including language skills, processing speed, attention, visuospatial abilities, and reduced abilities in EFs [59].

### 4.1. Executive Function and Motor Skills

Brain structure and function can impact the development of mental and motor abilities, while central nervous system (CNS) disorders may be linked to genetics. For example, individuals with DS often experience various brain-related issues that lead to delayed psychomotor development and learning difficulties [60]. In particular, the main issues affecting the CNS and resulting in psychomotor dysfunction in children with DS are changes in the number and shape of cerebrum cells and its size. Disorders of the CNS and pathophysiological processes encompass degenerative nervous system changes, dysregulated neuronal apoptosis, excessive expression of beta amyloid precursor protein coding genes, and mechanisms resulting in a reduced release of neurotransmitters [25].

Substantial alterations in cerebrum size and developmental setbacks manifest in individuals with DS after the sixth month of life [61]. Neuroimaging studies have demonstrated volumetric anomalies in various brain regions, including smaller frontal, occipital, and temporal lobes, reduced hippocampal volume, diminished corpus callosum and cerebellum size, and a decrease in superior temporal gyrus and brainstem volume [62]. These structural irregularities are associated with psychomotor impairments in individuals with DS, including difficulties in voluntary activities, cognitive deficits, and gait quality, particularly in adulthood. An MRI study revealed a decrease in granule cell density in children with DS, reducing it to around 70% compared to typically developing children [63]. This cerebellar hypoplasia contributes to muscle hypotonia, challenges in movement fluidity, issues with axial control (truncal muscle function), as well as difficulties in maintaining body balance, coordination, and speech [63]. Additionally, children with DS exhibit a reduction in corpus callosum size, which is linked to mental retardation, coordination problems, and atypical lateralization [63]. Indeed, cerebellar and corpus callosum that lead to hypoplasia are key contributors to muscle hypotonia, reducing movement fluency, compromised axial control, coordination difficulties, atypical lateralization, and impaired balance abilities [25]. Hypotonia, in turn, contributes to joint instability due to tendon laxity [25]. Furthermore, muscle weakness, disruptions in sensory integration processes, cartilage hypoplasia, and compromised bone density all contribute to inadequate muscle co-contraction [25]. In this regard, there is a 50% chance of independent standing at 2 years in children with DS, and most of them learn to stand between 3 and 4 years old [64]. Further, the prospect of climbing stairs, running, and jumping around 4 years of age is only 18–25%, and, by 6 years of age, the chance ranges between 65 and 85% [64].

Also, a reduction in grey matter volume within the cerebellum, frontal lobes, and the hippocampus, as well as a decrease in white matter volume in the left cerebellum and frontal and parietal lobes in individuals with DS explains the connection between low levels of EF outcomes and poorer motor skills, and vice versa [65]. Additionally, individuals with DS displayed abnormal sensorimotor integration or a compromised somatosensory system, which plays a crucial role in EF development [66].

### 4.2. EF Measures in DS Subjects

The prevalence of dementia in older DS patients is increasing; further, it is associated with high mortality rates [67]. In light of this, many studies tried to develop tests to assess the different aspects of cognitive performance and EFs in subjects with DS. Specifically, for everyday cognitive function, one of the most used was the Cognitive Scale for DS [58]. This informant-rated questionnaire evaluated daily abilities relating to EFs (such as memory and language, regardless of cognitive ability).

Further, starting in childhood, subjects with DS have a high probability of demonstrating a distinct cognitive profile characterized with deficits in verbal processing, working memory, and goal-directed behavior [68]. For this reason, another important test that is used in subjects with DS is the Behavior Rating Inventory of Executive Function (BRIEF). BRIEF is a questionnaire that consists in two versions (BRIEF-Adult, BRIEF-Children), and it is used to assess everyday EF behaviors. BRIEF is an assessment tool designed to evaluate executive skills in children and adolescents [69]. It can be used in a wide age range, usually from 5 to 18 years. There are versions adapted for younger and older ages, but it is designed is mainly designed for school-aged children and adolescents. However, it is important to note that adaptations and specific uses of the questionnaire may vary according to the norms and indications of the clinical or research context in which it is used.

Collins and Koechlin [70] show that EFs are fundamental to build higher-order EFs, such as reasoning, problem solving, and planning. For this reason, they are essential for mental and physical health and psychological and social development, particularly in subjects with DS where cognitive planning is commonly cited as a deficit. Indeed, cognitive planning is vital for a good quality of life (planning involved in grooming, dressing) and independence (managing steps involved in transportation, jobs) in subjects with DS [71].

A common test used to evaluate higher-order planning EFs in subjects with DS [32,72] was the Tower of London [73,74]. Administration of the Tower of London test in individuals with DS may vary depending on an individual’s specific cognitive and understanding abilities. However, in general, this test can be used with children or adolescents with DS [54,73,74]. Further, there exists a validated version test (Tower of London DX), which is mainly designed to be used with adults and adolescents from 16 years old and up. The DX (Delis–Kaplan Executive Function System) version of the test was developed to evaluate executive functions in adults [75]. The Tower of London test comprises two wooden peg boards (one each for the participant and evaluator) with three vertical pegs of different lengths and three beads (one green, blue, and red). Participants were asked to recreate the demonstrated configuration of beads on the peg board.

Further, Edgin et al. [76] showed that the Cambridge Neuropsychological Test Automated Battery (CANTAB) is a good instrument to evaluate working memory: specifically, it measured visuospatial memory and information processing speed (reaction time). The CANTAB consists of various tests designed to evaluate different cognitive domains, including memory, attention, executive function, and visual perception. The CANTAB battery includes an evaluation on spatial working memory, assesses spatial working memory and strategy utilization, evaluates visual memory and new learning abilities with paired associates learning, measures cognitive flexibility and the capacity for attentional set shifting with an attention switching task, assesses both simple and choice reaction time with reaction time, evaluates verbal memory and recognition skills with verbal recognition memory, assesses motor speed and coordination with a motor screening task, measures rule acquisition and the ability for reversal learning with an intra-extra dimensional set shift, and evaluates visual memory and the capability for pattern recognition with delayed matching to a sample. The CANTAB test can be administered to individuals from 4 to 64 years of age [77,78]. In children, improvements were observed in the efficiency of working memory capacity, planning, and problem-solving abilities. The CANTAB has been reported to be highly applicable for evaluating children with limited verbal skills, such as those associated with learning disabilities, autism, or both.

Another popular test to evaluate inhibition was NEPSY II [79,80]. NESPY II includes 32 individual subtests and 4 delayed recall subtests. The wide range of NEPSY-II subtests is designed to assess neuropsychological functioning across six domains, including attention and EFs, visuospatial processing, sensorimotor, memory, and learning skills, social perception, and language skills [81]. The data reported in the manual suggest that NEPSY-II scores have good discriminative validity across a variety of disability conditions [82].

NEPSY-II is designed for individuals aged 3 to 19 [79,82].

Friedman et al. [83] suggest that shifting involved strategic planning and flexibility of thought and action. Indeed, EF deficits have been found in people with intellectual disability. Particularly, DS exhibits impairment in conceptual shifting, set shifting, and those deficits which are age-related and appear associated with the onset of early dementia [53]. In view of the importance of shifting in everyday life of DS, it becomes fundamental to monitor the shifting in research that embrace subjects with DS.

The Intra-Extra Dimensional Set Shift (IED) test, one of most used tests to evaluate shifting, was proposed by Edgin et al. [76]. IED is a component of the CANTAB. This test assesses cognitive flexibility, rule acquisition, and reversal learning. It is often used to evaluate executive function and cognitive skills in various populations, including individuals with DS [66]. The IED test can be administered in subjects from 4 to 64 years of age [77].

Moreover, Zelazo et al. [84] proposed the Dimensional Change Card Sort (DCCS) test to assess shifting.

The DCCS test is used to assess cognitive flexibility, attention shifting, and EFs in individuals, including children. The test is often used in neuropsychological assessments, developmental psychology, and research studies [84]. The DCCS test typically involves a card-sorting task where participants are required to sort cards based on changing rules or factors. Initially, participants have to sort cards based on one factor (color). After this, the rules change, and participants are instructed to sort the cards based on a different factor (shape). This shift in rules assesses the individual’s ability to flexibly adapt thinking and cognition [84]. The DCCS test is commonly used in assessing cognitive development in children, particularly those between the ages of 3 and 7 years [76].

### 4.3. Gender Differences in MS in Subjects with DS

Children with DS tend to have difficulties with fine motor skills due to their low muscle tone and/or hypermobility in their hands, wrist, and/or elbows. However, current literature evidence shows that children and adolescents with DS display differences in motor performance precision because of their sex [85]. Traditionally, males showed a prevalence in gross motor performance and females a prevalence in fine motor performance [25]. However, the severity of DS is associated with intellectual performance, communication difficulties, and self-sufficiency [86]. In fact, most of the subjects with DS have learning-related problems, depending on age and gender, that are significantly associated with the severity of DS. As shown previously in numerous studies, the physical fitness of participants with DS was lower compared to their peers. According to Schields et al., the reasons for non-participation in physical activity by children with disabilities are complex and multifactorial [32]. A range of personal, social, environmental, and policy- and program-related barriers and facilitators influence the amount of PA that children with disabilities perform. In fact, children with intellectual and physical disabilities are less active than their typically developing peers [87]. For these reasons, an increased awareness of DS phenotypic profiles among professionals and caregivers can foster earlier detection and counselling and help formulate appropriate interventions to reduce long-term sequelae and enhance cognitive and behavioral developmental outcomes [87]. Youth with physical disabilities are also reported to have less variety in their recreation and leisure participation, spending more time in sedentary recreational activities than their typically developing peers and in slower-tempo skills-based activities and sports [88] Therefore, since children with disabilities often have lower levels of PA, understanding motivation is a crucial element that has to be taken into consideration to enhance PA participation. In relation to this, the study of Barr et al. highlighted the role of families in determining to what extent PA in children with DS and the effect that common characteristics associated with DS could have on maintaining an active lifestyle. Furthermore, children’s compliance is prominent for successful interventions, and is linked to school readiness and later social and behavioral competence [86].

### 4.4. Training of EFs in Subjects with DS

There is a limited number of studies that investigated physical training effects on EFs in subjects with DS. Despite this, different studies proved that aerobic exercise could improve physical performance and EFs in subjects with DS.

For example, Ringenbach et al. [28] suggested that cycling (assisted, voluntary) could improve EFs in subjects aged around 18 years with DS. Specifically, after eight weeks (cycling, 3 times per week), the cycling groups improved more on the shifting than the control group; further, the cycling groups improved significantly more than the control group in the language fluency score. These results lead the authors to affirm that light aerobic activity can benefit set-shifting ability.

Ringenbach et al. [28] implemented their research to compare resistance and aerobic training and to establish which type improves EFs more in adults with DS. Specifically, they divided subjects with DS into 3 different groups: (i) resistance training, (ii) assisted cycle therapy, and (iii) no training. A total of 14 adults with a mean mental age of 6.18 years were involved in the study. The results showed that inhibition time improved following assisted cycle therapy and resistance training; however, it varied only between the assisted cycle therapy and no training groups [28,89]. Furthermore, the authors suggest that another explanation for the increase of EFs in the assisted cycle therapy group was the level of intensity of cycling, embracing the hypothesis which states that lower and higher exercise intensities will not have as much of an effect on EFs in comparison to moderate-intensity exercises.

Moreover, Chen et al. [89] analyzed the effect of a single bout of moderate physical activity on the EFs in young adults with DS. The protocol compared 32 subjects with a mental age lower than 3 years, in which they were divided into the following two groups: (i) intervention group (walking on a treadmill) and (ii) control group (watching a movie). The protocol consisted of a single bout of training which lasted 20 min. The results showed that adults with DS improved cognitive performance in inhibition after a 20 min single bout of treadmill walking at moderate intensity (i.e., 65% of their predicted max heart rate). However, the precise underlying mechanism for this increase in individuals with DS is still unclear. Based on previous studies linking exercise and EFs, the possible explanation for this is described by the arousal theory [90]. Further, the authors suggested that another possible explanation is the increased expression of neurotrophins, such as the brain-derived neurotrophic factor [91] and elevated blood flow (glucose level) in certain brain areas [92] following exercise. In accordance with previous studies just described [28,72,93], moderate-intensity physical activity seems to be a good alternative to improve EFs in subjects with DS.

Another type of activity that was proven to be efficient in subjects with DS was the dual-task activity. The dual-task activity consisted of performing two tasks simultaneously, such as walking and holding a plate and cup, or carrying a tray and cup.

As shown by Latash [94], dual-task activities, when in line with the use of basic principles of motor control and learning, can greatly improve the motor synergies and motor performances of subjects with DS aged 18–28 years [95].

In view of this, Horvat et al. [95] analyzed the effect of the dual-task protocol in 12 individuals (18–28 years) with DS and 12 individuals without disabilities. Both groups were matched according to age, gender, and activity participation. The groups were divided as follows: (i) dual-task group: walking with cognitive tasks; (ii) control group: walking alone. The results showed that subjects with DS were less efficient and more variable in their gait performances than young adults without disabilities. Further, Horvart [96] suggested that the less a motor program is developed, the harder it is for a person to control their movement.

Shields et al. [88] created the FitSkills program for adolescent (13–17 years) with DS, which consisted of an incremental protocol of resistance and aerobic training designed for DS [97].

Recently, this protocol was tested on 20 adolescents and young adults with DS (aged 13 to 35 years, two times per week, for twelve weeks) to evaluate their EFs, and the results were compared with a control group. The findings showed differences in the measures of verbal and nonverbal intelligence in favor of the experimental group; however, no differences were found in EFs for everyday tasks, such as planning, inhibitory control, or attention-shifting assessments. Further, no differences were found between the groups in relation to their working memory and processing speeds. In summary, this study provides an effective protocol for improving everyday EFs in young people with DS.

## 5. Conclusions

In conclusion, motor skills interventions are effective in improving motor competence and performance in cognitive, emotional, and physical aspects of children with DS according to their stage of developmental. During the first years of life, the connection between motor and mental development is very strong. In fact, children obtain knowledge about the world through touching, taking toys into their mouths, and observing [31]. Movement is considered the basis for learning new abilities (even cognitive abilities) in the first years of life. Indeed, the age at which children with DS achieve GMS is at approximately twice the age of children that have developed in a typical manner [25]. For this reason, improvements in functions include minimized vulnerability to fractures, dislocations, and complications resulting from hypermobility and motor acquisitions, which can be achieved in an adequate amount of time [41].

Based on the previous studies, EFs increased even after (only) a single bout of 20 min of moderate physical activity. Indeed, all protocols suggested that moderate-intensity physical activity was the optimum activity level for improving EFs in subjects with DS. Indeed, Kasari et al. [90] suggested that the possible reason could be described by the arousal theory. Further, every protocol evaluates EFs in less than one hour per lesson. Horvart et al. [95,96] suggested the importance to play with locomotor skills, including dual tasks, which is in line with Falbo et al. [98] who showed that variations in inhibition performance were correlated with the changes in dual-task walking performance in older adults, improving gait performance. Other aerobic activities, even resistance training (8–12 repetitions at 75% of the participant’s 1RM), could seem to improve EFs. Other studies on subjects with DS have used a protocol based on resistance training and demonstrated an increase in balance and strength [37], cognitive function [99], motor behavior, mood, and physical fitness [100] without investigating EFs. In summary, interventions based on EFs, which are tailored to the age of subjects with DS, are effective in contrasting the cognitive decline and showing improvements in EFs in young and adults. The implementation of targeted accessible programs and recreation centers supported by governments and private sectors can help to promote PA participation of subjects with DS, improving overall health outcomes and QoL. 

## Data Availability

Not applicable.
